# The later-line efficacy and safety of immune checkpoint inhibitors plus anlotinib in EGFR-mutant patients with EGFR-TKI-resistant NSCLC: a single-center retrospective study

**DOI:** 10.1007/s00262-024-03712-7

**Published:** 2024-05-17

**Authors:** Xiaoyan Yin, Xinchao Liu, Fei Ren, Xiangjiao Meng

**Affiliations:** Department of Radiation Oncology, Shandong Cancer Hospital and Institute, Shandong First Medical University and Shandong Academy of Medical Sciences, Jinan, Shandong China

**Keywords:** Non-small cell lung cancer, EGFR-TKI resistance, Immune checkpoint inhibitor, Anlotinib, Efficacy, Safety

## Abstract

**Background:**

Effective treatment after EGFR-TKI resistance is of great clinical concern. We aimed to investigate the efficacy and safety of anlotinib in combination with an anti-PD-1/PD-L1 antibody in later-line therapy for EGFR-mutant NSCLC patients after TKI treatment failure and to explore the independent predictive factors of therapeutic efficacy.

**Methods:**

A total of 71 patients with confirmed advanced *EGFR*-mutated NSCLC who progressed after previous standard EGFR-TKI therapy but still failed after multiline treatments were included retrospectively in this study. Most of the patients had previously received at least three lines of treatment. All were treated with anlotinib combined with anti-PD-1 or anti-PD-L1 therapy. The safety of this combined treatment was assessed by the incidence of adverse events. The efficacy of the regimens was evaluated by survival analysis (OS, PFS, ORR, DCR).

**Results:**

The median follow-up period was 28.6 months (range: 2.3–54.0 months), and the median number of treatment lines was 4. The overall response rate (ORR) and disease control rate (DCR) were 19.7% and 77.5%, respectively. The median PFS was 5.8 months (95% CI 4.2–7.4 months), and the median OS was 17.1 months (95% CI 12.0–22.3 months). Patients who received immune checkpoint inhibitors plus anlotinib had an encouraging intracranial ORR of 38.5% and a DCR of 80.8%. ECOG performance status < 2 at baseline was independent protective factors of PFS. Metastatic organs and ECOG performance status were independent parameters in predicting OS. Treatment-related adverse events occurred in 66 (93.0%) patients; most of the adverse events were Grade 1–2, and no increase in adverse events was observed compared to monotherapy.

**Conclusion:**

Anlotinib combined with an anti-PD-1/PD-L1-based regimen exhibited promising efficacy and tolerance in NSCLC patients with EGFR mutations after previous TKI failure. The efficacy of this combined regimen in patients with EGFR mutations should be further evaluated.

**Supplementary Information:**

The online version contains supplementary material available at 10.1007/s00262-024-03712-7.

## Background

Epidermal growth factor receptor (EGFR) mutations are the most important driver mutations in lung cancer. The mutation rate of EGFR in Asian lung adenocarcinoma (LUAD) is as much as 50% [1, 2]. Personalized EGFR tyrosine kinase inhibitor (EGFR-TKI) targeted therapy is currently the standard first-line treatment for patients with advanced EGFR-mutant NSCLC, but drug resistance is still inevitable [3]. This highlights the need for a new strategy in the clinic for patients who experience disease progression after developing EGFR-TKI resistance. Currently, many ICI antibody monotherapies or combined therapies, such as pembrolizumab, nivolumab, and atezolizumab, have been approved by the Food and Drug Administration for clinical application in lung cancer [4–6]. However, immunotherapy in NSCLC patients with EGFR mutations is still controversial. Because the immunogenicity of EGFR-mutant NSCLC is weak, while CD8 + T-cell infiltration decreases, antitumor effects weaken, Treg cells increase, and CD37 is overexpressed; all of the above findings indicate that EGFR-mutant NSCLC has an immunosuppressive tumor microenviroment (TME), which renders these NSCLC patients insensitive to immunotherapy [7, 8]. Several studies demonstrated that a lower proportion of PD-L1-positive cells was present in NSCLC patients harboring EGFR mutations than in those harboring wild-type PD-L1, revealing a negative correlation between EGFR mutation status and PD-L1 expression (*P* = 0.003) [9]. Thus, the benefits of immunotherapy alone are very limited. Fortunately, the use of immune checkpoint inhibitors combined with antiangiogenic therapy initially has shown a positive effect.

According to the key subgroup analysis of the IMPOWER 150 study, bevacizumab could improve survival in patients with EGFR-mutant NSCLC via combination with immunotherapy plus chemotherapy [10, 11]. In addition, ORIENT-31 further confirmed the efficacy of combined immunotherapy (sintilimab with bevacizumab plus chemotherapy) in the EGFR-TKI resistant population [12, 13]. However, there are still some aspects of this regimen that do not meet clinical needs. A high incidence of grade 3 or 4 treatment-related adverse events could be reached [14, 15]. Furthermore, patients who cannot tolerate chemotherapy should be considered and the efficacy of multiple lines treatment is uncertain. Anlotinib, which is a novel multitargeted tyrosine kinase receptor inhibitor that functions by acting fully on vascular endothelial growth factor receptor (VEGFR)1–3, fibroblast growth factor receptor (FGFR)1–4, platelet-derived growth factor receptor (PDGFR)α/β, c-Kit, Met and Ret [16–18] to reprogram the TME and enhance the efficacy of immunotherapy, has been approved as a third-line therapy in advanced NSCLC patients by the Chinese Food and Drug Administration (CFDA). In the ALTER0303 trial, anlotinib was well tolerated as third-line or further therapy and significantly improved PFS and OS in both EGFR-mutated and EGFR-wild-type patients with advanced NSCLC [19]. The use of anlotinib combined with immunotherapy has been preliminarily reported in wild-type NSCLC patients [14, 17, 20, 21], but efficacy in advanced NSCLC patients harboring mutations has not been determined. Therefore, further research is needed to prove the antitumor effect of combined drugs in the clinic.

This retrospective study investigated the safety and efficacy of anlotinib plus immune checkpoint inhibitor as a later-line therapy in EGFR-mutant NSCLC patients.

## Methods

### Study design and patients

**Figure1:**
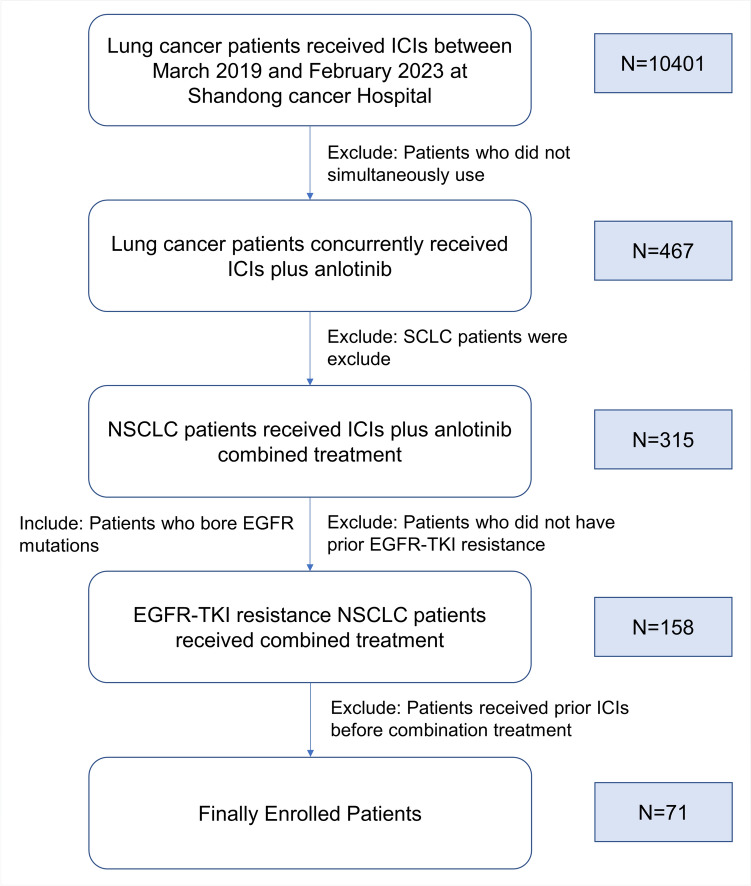
CONSORT diagram. NSCLC, non-small cell lung cancer; SCLC, small cell lung cancer; ICI, immune checkpoint inhibitor; EGFR, epidermal growth factor receptor; TKI, tyrosine kinase inhibitor

This retrospective study was designed to evaluate the safety and efficacy of combination therapy comprising anlotinib and ICIs in patients with advanced NSCLC harboring EGFR mutations. Patients were enrolled between March 2019 and February 2023 at Shandong Cancer Hospital Affiliated with Shandong First Medical University (Fig. 1). Follow-up data were collected through September 20, 2023. The inclusion criteria included histologically or cytologically confirmed advanced-stage (IIIB-IV) NSCLC, the genetic test shown EGFR mutations, an Eastern Cooperative Oncology Group (ECOG) PS score of 0–2 and previous treatment with EGFR-TKIs with or without chemotherapy. The exclusion criteria included immeasurable tumor lesions, concomitant other cancers, and prior immunotherapy. Patients with EGFR mutations were required to have disease progression with at least one approved TKI therapy. The 22C3 pharmDx assay (Agilent Technologies) was used to assess PD-L1 expression, and PD-L1 positivity (PD-L1 +) was defined as a PD-L1 tumor proportion score (TPS) ≥ 1%. This study was conducted in accordance with Good Clinical Practice guidelines and the Declaration of Helsinki and was authorized by the Institutional Review Board of Shandong Cancer Hospital affiliated with Shandong First Medical University. The requirement for informed consent was waived in this retrospective analysis.

### Treatment

We retrospectively enrolled patients with EGFR-mutant advanced NSCLC who experienced disease progression after treatment with EGFR-TKIs. All patients in this study received anlotinib combined with anti-PD/PD-L1 antibody treatment. Patients received intravenous anti-PD-(L)1 agents at a dose of 200 mg once on day 1, followed by subsequent administrations every 3 weeks, and anlotinib (Chia Tai Tianqing Pharmaceutical Group Co., Ltd.) was orally administered once a day (8 mg, 10 mg or 12 mg) for 14 days within a 21-day cycle. The combined agents were continued until disease progression or appearance of unacceptable toxicity. Target lesions were evaluated using computed tomography (CT) or magnetic resonance imaging (MRI) for each patient before and during the combination treatment.

### Outcomes

Radiological assessments of tumor lesions were performed every two cycles during treatment. Tumor response was evaluated by the Response Evaluation Criteria in Solid Tumors version 1.1 (RECIST v1.1). Tumor responses included complete response (CR), partial response (PR), stable disease (SD), and progressive disease (PD). Progression-free survival (PFS) was defined as the time from the first use of ICIs and anlotinib to disease progression or mortality from any cause. Overall survival (OS) was defined as the time during the period of the first-time taking ICIs and anlotinib to the last follow-up or mortality. The overall response rate (ORR) was defined as the ratio of patients who achieved CR plus PR for target and metastatic lesions. The disease control rate (DCR) included the proportion of patients who achieved a CR, PR or SD among the whole population of tumor patients receiving treatment. The intracranial ORR (iORR) was calculated based on brain lesions and was assessed by an independent, blinded radiologist according to the RECIST v1.1. The longest diameter of the target lesions was at least 10 mm, and a maximum of two target brain lesions were tracked. Treatment-related adverse events (TRAEs) were evaluated and graded by the Common Terminology Criteria for Adverse Events (CTCAE) version 5.0.

### Statistical analyses

Descriptive data were used to represent demographic and clinical variables. We utilized the Kaplan–Meier method to evaluate survival. Univariate and multivariate Cox regressions were performed, simple correlation analysis was performed for comparisons through univariate regression, and multivariate Cox regression was subsequently used to explore the influence of multiple factors on survival outcomes. Variables with *p* values < 0.1 in the univariate analysis were selected for analysis via the multivariate Cox regression model. All statistical tests were two-sided, and *p* value < 0.05 was considered to indicate statistical significance. The statistical analysis was carried out with PRISM version 8.0.2 (GraphPad Software, La Jolla, CA, USA) and SPSS version 26.0 (IBM Corp., Armonk, NY, USA).

## Results

### Patient characteristics

A total of 71 patients were enrolled in this retrospective study, and the related demographic and clinical characteristics are listed in Table 1. The median age was 55 years (range: 33–73 years). The median number of treatment lines was 4 (range: 2–8), and 59.2% of patients had received previous third- or further-line systemic therapy. Patients with ECOG performance status 0–1 and 2 accounted for 81.7% and 18.3%, respectively. The sensitive mutations of EGFR exon 21 L858R and 19 exon del accounted for 35.2% (25 patients) and 49.3% (35 patients), and rare mutations only accounted for 15.5% (11 patients). In addition, 30 patients (42.3%) had acquired Thr790Met (T790M) mutations. 27 patients (38.0%) previously received first- or second-generation EGFR-TKIs, and 37 patients (52.1%) developed resistance to first- or second-generation TKIs and received third-generation EGFR-TKIs. A total of 11, 29, and 31 patients were given 8, 10 and 12 mg of anlotinib, respectively. Liver, bone or brain metastases were present in 15 (21.1%), 40 (56.3%), and 30 (42.3%) patients. More than half of the patients had ≥ 3 metastatic sites (57.7%). Of the 30 patients with brain metastasis, 19 patients received intracranial radiotherapy, of which 18 patients received whole brain radiation therapy (WBRT) and 1 patient received stereotactic radiosurgery (SRS). A total of 18 patients (25.4%) reported PD-L1 expression at baseline statistics, of which 14 patients (19.7%) were PD-L1 positive (TPS ≥ 1%) and 4 patients (5.6%) were PD-L1 negative (TPS < 1%). Before administration of combined treatment, 8 patients (44.4%) evaluated PD-L1 expression and 10 patients (55.6%) received PD-L1 detection simultaneously during diagnostic puncture biopsy (i.e. synchronous with genetic testing). 53 patients (74.6%) were treated with ICIs plus anlotinib, 18 (25.4%) patients received chemotherapy in combination with ICIs plus anlotinib, and most of them were treated with ICIs combined with single-agent chemotherapy. The corresponding agents were gemcitabine, pemetrexed or paclitaxel. Camrelizumab (20 patients, 28.2%) and sintilimab (38 patients, 53.5%) were the two main anti-PD-1 drugs. A total of 7.1% of patients received tislelizumab (5/71). Furthermore, toripalimab, nivolumab, pembrolizumab and atezolizumab were administered to two patients, accounting for 11.3% of the total patients.

**Table 1:** Clinical and pathological characteristics of the patients

Factor	*n* (%) Total *n* = 71
Sex	
Male	28 (39.4)
Female	43 (60.6)
Age, median (range, years)	55 (33–73)
Histological subtype	
Adenocarcinoma	65 (91.5)
Squamous and others*	6 (8.5)
Smoking history	
Never smoked	59 (83.1)
Former/current smoker	12 (16.9)
ECOG performance status	
0–1	58 (81.7)
2	13 (18.3)
Type of mutation	
EGFR exon 19 del	35 (49.3)
EGFR exon 21L858R	25 (35.2)
Rare mutations	11 (15.5)
Acquired T790M mutation	
No or Unknown	41 (57.7)
Yes	30 (42.3)
Prior EGFR-TKI therapy	
1st or 2nd-generation TKIs	27 (38.0)
3rd-generation TKIs	7 (9.9)
1st or 2nd + 3rd-generation TKIs	37(52.1)
Combined chemotherapy	
No	53 (74.6)
Yes	18 (25.4)
No. of metastatic organs	
< 3	30 (42.3)
≥ 3	41 (57.7)
Brain metastases	
Absent	41 (57.7)
Present	30(42.3)
Liver metastases	
Absent	56 (78.9)
Present	15 (21.1)
Bone metastases	
Absent	31 (43.7)
Present	40 (56.3)
Anlotinib dosage	
8 mg	11 (15.5)
10 mg	29 (40.8)
12 mg	31 (43.7)
PD-L1 expression	
> 50%	5 (7.0)
1–50%	9 (12.7)
Negative* or unknown	57 (80.3)
Types of anti-PD-(L)1	
Sintilimab	38 (53.5)
Camrelizumab	20 (28.2)
Others	13 (18.3)
PD-1 vs. PD-L1 Inhibitors	
PD-L1	2 (2.8)
PD-1	69 (97.2)
Combined therapy lines	
< 4	29(40.8)
≥ 4	42 (59.2)
Median (range, lines)	4 (2–8)

*Others refer to adenosquamous carcinoma and one case of adenocarcinoma transformed into small cell lung cancer; PD-L1 negative was defined as PD-L1 tumor proportion score (TPS) < 1%; ECOG-PS, Eastern Cooperative Oncology Group Performance Status; EGFR, epidermal growth factor receptor; PD-L1, programmed cell death ligand-1; PD-1, programmed cell death-1; TKI, tyrosine kinase inhibitor

### Efficacy

**Fig. 2:**
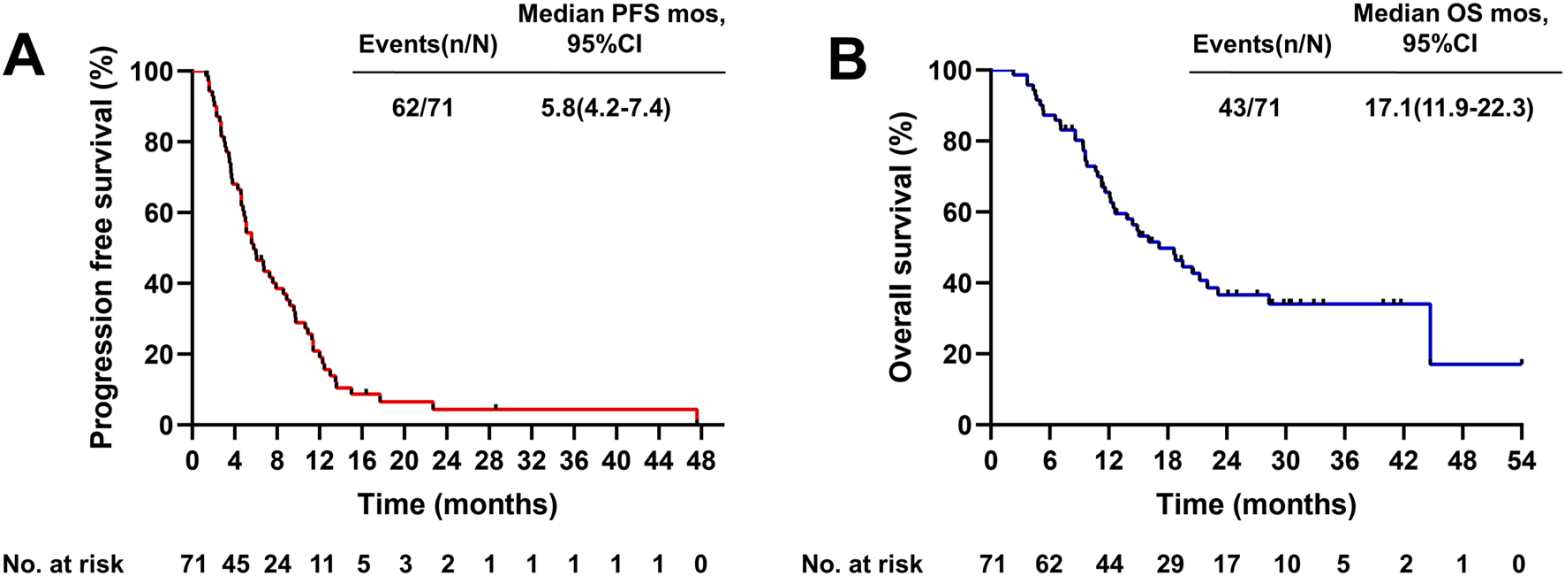
Survival outcomes **A** progression-free survival; **B** overall survival; Mo, months; CI, confidence interval

**Fig. 3:**
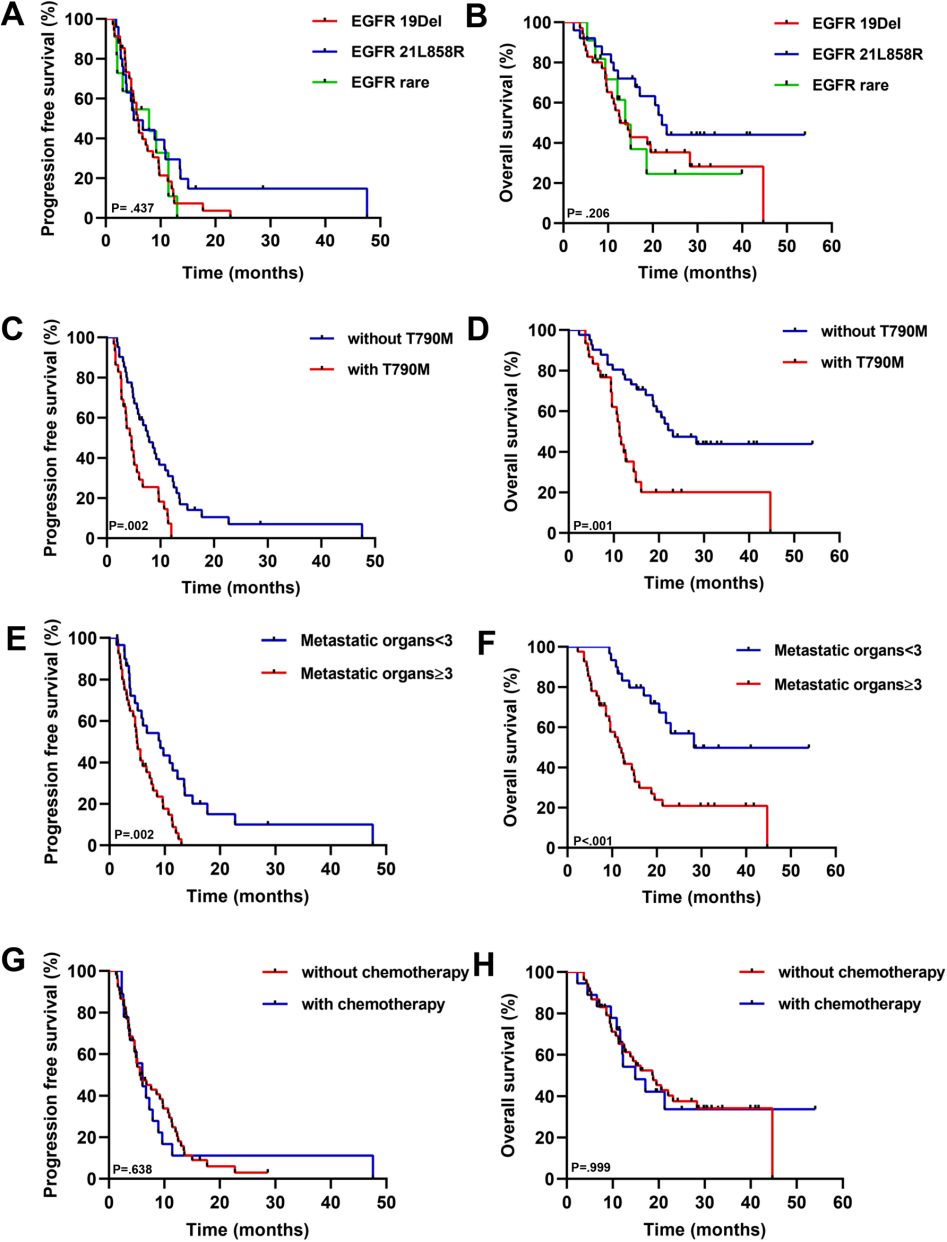
Survival outcomes in key subgroups **A** PFS of patients with different EGFR mutation types; **B** OS of patients with different EGFR mutation types; **C** PFS of patients with or without acquired T790M; **D** OS of patients with or without acquired T790M; **E** PFS of patients with different numbers of metastatic organs; **F** OS of patients with different numbers of metastatic organs; **G** PFS of patients with chemotherapy or without chemotherapy; **H** OS of patients with chemotherapy or without chemotherapy; PFS, progression-free survival; OS, overall survival

**Fig. 4:**
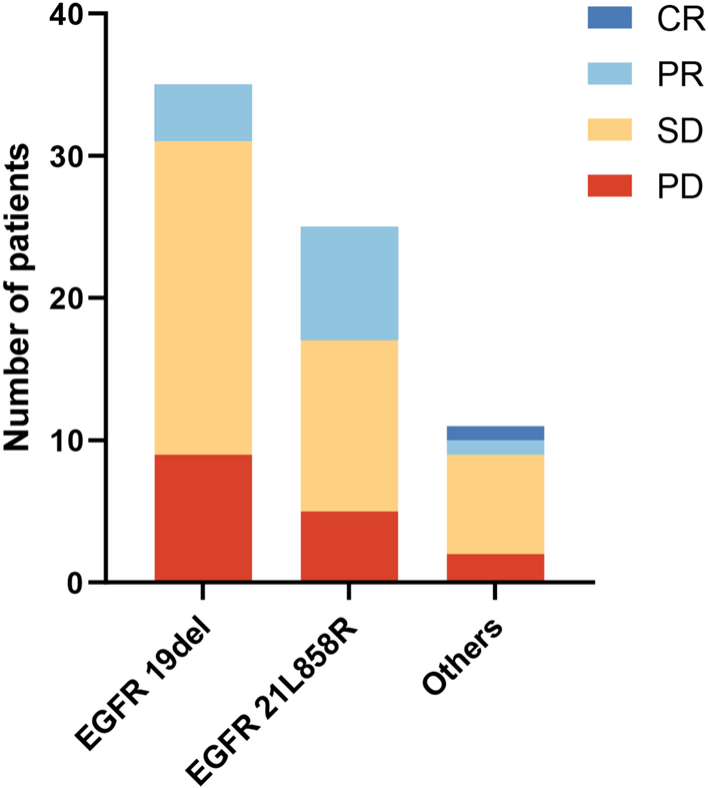
Tumor response in patients with different types of EGFR mutations. EGFR, epidermal growth factor receptor; CR, complete response; PR, partial response; SD, stable disease; PD, progressive disease

**Fig. 5:**
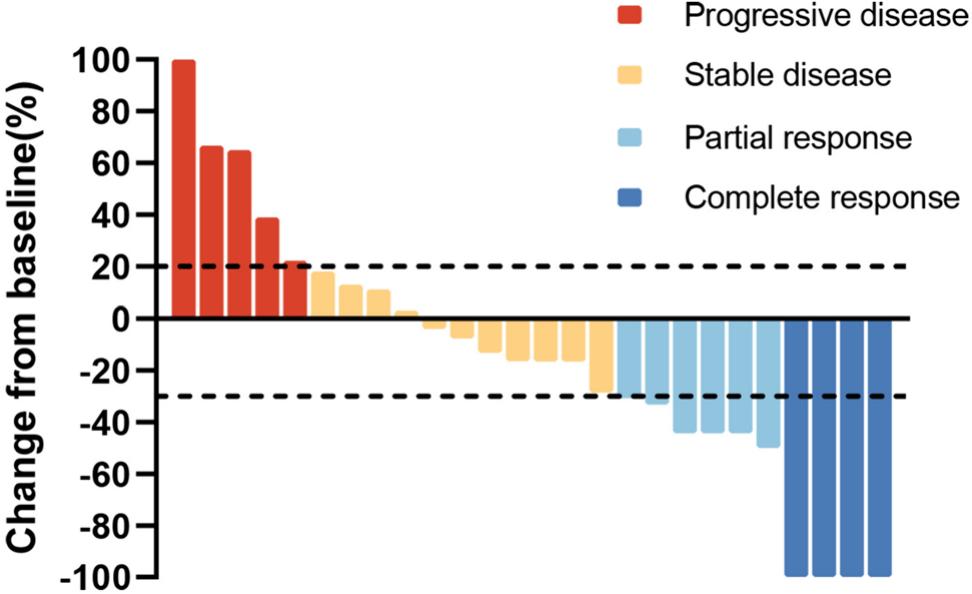
Waterfall plot of the optimal percentage change in tumor lesion size. Among the 30 patients with brain metastases, 26 patients had complete imaging data available, revealing the greatest percentage change in target lesion size; among them, 4 were assessed as complete response, 6 were assessed as partial response, 11 remained stable and 5 had disease progression

The median follow-up period was 28.6 months (range: 2.3–54.0 months). One patient (1.4%) achieved complete response (CR), 13 patients (18.3%) achieved PR, 41 patients (57.8%) achieved SD, and 16 patients (22.5%) developed progressive disease (PD), yielding an ORR of 19.7% and a DCR of 77.5% (Table 2). The median PFS was 5.8 months (95% CI 4.2–7.4 months), and the median OS was 17.1 months (95% CI 12.0–22.3 months) (Fig. 2, Table 2). Patients harboring EGFR 21L858R mutation had longer OS (mOS, 22.0 months) than those harboring the EGFR 19del mutant (mOS, 12.6 months) or the EGFR rare mutant (mOS, 13.8 months); however, the rare subgroup presented better PFS (mPFS, 7.9 months), and the mPFS of the EGFR-sensitive mutant was no more than 6 months (Fig. 3A, B), but these differences were not statistically significant. The tumor response of patients with different EGFR mutations is shown in Fig. 4. In addition, in the survival analysis, for acquired resistance, we found that patients who bore acquired Thr790Met or less than three organ metastases showed distinctly longer survival (mPFS 7.9 months, mOS 23.1 months vs. mPFS 4.6 months, mOS 11.3 months; mPFS 9.2 months, mOS 28.3 months vs. mPFS 5.0 months, mOS 11.6 months) (*p* value < 0.05) (Fig. 3C, D; Fig. 3E, F). We also observed that the mPFS and mOS of the chemo-free patients were 5.6 months and 18.8 months, respectively. The mPFS and mOS of patients treated with chemotherapy combined with anlotinib plus ICIs reached 6.0 months and 14.9 months, respectively. However, the PFS and OS curves both intersected at the time of analysis, showing no statistical difference (Fig. 3G, H). Based on key subgroup analyses, patients who had liver metastases, brain metastases or bone metastases had the median OS of 10.7 months, 14.4 months and 14.9 months, respectively, and the median PFS were 5.6 months, 5.6 months and 5.1 months, respectively. As shown in the waterfall plot (Fig. 5), 26 of the 30 patients with brain metastases had unabridged imaging data and measurable intracranial lesions, 4 patients (15.4%) achieved iCR, 6 patients (23.1%) exhibited iPR, 11 patients (42.3%) had iSD, and 5 patients (19.2%) developed iPD, yielding the intracranial overall response rate (iORR) of 38.5% and the intracranial disease control rate (iDCR) of 80.8%. Notably, one of the iCR patients was given intrathecal injections of methotrexate along with systemic administration of ICI plus anlotinib. Additionally, Patients who received brain radiotherapy at baseline showed longer survival tendency than those who had not received intracranial RT (OS: 11.6 mos vs. 15.1mos), but no statistical difference (*p* = 0.403). For patients with measurable brain metastases and complete imaging information, we found that the iORR of patients who without intracranial radiotherapy (iRT) and those who had radiotherapy were 44.4% and 35.3%, and the iDCR was 77.8%, 88.4%, respectively.

**Table 2:** Efficacy of Anlotinib combined with an anti-PD1/PD-L1 antibody in later-line treatment of NSCLC patients

Efficacy	All patients (*n* = 71)
Complete response, *n* (%)	1 (1.4)
Partial response, n (%)	13 (18.3)
Stable disease, *n* (%)	41 (57.8)
Progressive disease, *n* (%)	16 (22.5)
ORR (%, CR + PR)	19.7
DCR (%, CR + PR + SD)	77.5
mPFS (months) (95% CI)	5.8 (4.2–7.4)
mOS (months) (95% CI)	17.1 (11.9–22.3)
iORR (%, CR + PR)	38.5
iDCR (%, CR + PR + SD)	80.8

*CR* complete response, *PR* partial response, *SD* stable disease, *PD* progressive disease, *iORR* intracranial objective response rate, *iDCR* intracranial disease control rate, *mOS* median overall survival, *mPFS* median progression-free survival

We further performed Cox regression analysis to explore potential features that were associated with the prognosis of combination therapy. According to univariate Cox analysis, the p values for ECOG-PS, metastatic organs, T790M mutation and bone metastases were < 0.1 and indicated association with PFS (*p* = 0.002, *p* = 0.002, *p* = 0.003, *p* = 0.076, respectively); additionally, the ECOG-PS (*p* < 0.001), metastatic organs (*p* < 0.001), T790M mutation (*p* = 0.001), type of EGFR mutation (*p* = 0.096) and liver metastases (*p* = 0.010) were associated with OS (Table 3). In multivariate analysis, we observed that ECOG-PS were significantly associated with PFS and OS. ECOG-PS 0-1 showed better survival to the combined treatment (PFS: *p* = 0.049, HR = 0.493, 95% CI = 0.244-0.998; OS: *p* = 0.009, HR = 0.368, 95% CI = 0.147-0.777). Additionally, metastatic organs were also found to independently predict OS. Patients with < 3 metastatic organs perhaps associated with a better prognosis (OS: *p* = 0.047, HR = 0.435, 95% CI = 0.207–0.989) (Table S1 in supplemental material).

**Table 3:** Univariate Cox regression analysis of factors associated with PFS and OS

Variables	PFS		OS	
	HR (95% CI)	*p* value	HR (95% CI)	*p* value
Gender				
Male Vs Female	0.905(0.536–1.528)	0.708	1.559(0.845–2.874)	0.155
Smoking status				
Never smoke Vs smoker	1.151(0.596–2.224)	0.675	0.899(0.414–1.955)	0.789
ECOG-PS				
0-1 Vs ≥ 2	0.350(0.180–0.681)	**0.002**	0.227(0.114–0.453)	** < 0.001**
Metastatic organs				
< 3 Vs ≥ 3	0.408(0.231–0.723)	**0.002**	0.303(0.155–0.593)	** < 0.001**
T790M mutation				
No Vs Yes	0.436(0.253–0.752)	**0.003**	0.360(0.192–0.673)	**0.001**
Type of mutation				
Rare Vs 19Del	1.024(0.501–2.092)	0.948	1.020(0.436–2.384)	0.963
21L858R Vs 19Del	0.700(0.392–1.250)	0.228	0.559(0.281–1.108)	**0.096**
Anlotinib dosage				
12 mg Vs 8 mg	1.147(0.554–2.372)	0.712	1.011(0.440–2.325)	0.979
10 mg Vs 8 mg	0.680(0.319–1.446)	0.316	0.809(0.345–1.898)	0.626
Liver metastases				
Absent Vs Present	0.602(0.327–1.106)	0.102	0.414(0.212–0.806)	**0.010**
Bone metastases				
Absent Vs Present	0.626(0.373–1.050)	**0.076**	0.805(0.436–1.487)	0.489
Brain metastases				
Absent Vs Present	0.810(0.483–1.359)	0.426	0.610(0.332–1.119)	0.111
Combined Chemotherapy				
No Vs Yes	0.871(0.490–1.550)	0.639	1.000(0.502–1.990)	0.999

*PFS* progression-free survival, *OS* overall survival, *ECOG-PS* Eastern cooperative oncology group performance status, *EGFR* epidermal growth factor receptor, *T790M* Thr790Met, *21L858R* 21Leu858Arg, *19Del* Exon 19 deletion, *HR* hazard Ratio, *Cl* confidence interval

In Univariate analysis, variables with *p* value < 0.1 were selected for analysis via the multivariate Cox regression model

Bold values indicates significant results with *p* < 0.1

### Safety

The overall incidence of adverse events (AEs) was 93.0% (66/71), most of the adverse events were Grade 1–2 (Table S2 in supplemental material), and no Grade 5 fatal adverse events were observed. 14 patients (19.7%) decreased one dose gradient of anlotinib under the guidance of clinicians, of which 6 patients (8.4%) had their dose adjusted to 10 mg/d and 8 patients (11.3%) adjusted to 8 mg/d. But, 5 patients (7.1%) stopped taking their drugs due to cardiac insufficiency, leukoderma (irAE), hand-foot syndrome, oral ulcers or grade 4 gastrointestinal reactions. For patients treated with anlotinib plus ICIs, the five most common adverse events (any grade) were fatigue, hypertension, transaminitis, hypercholesterolemia and nausea/vomiting, which were flexible to deal with and reversible. The most common treatment-related adverse events of grade 3 or more were hypertension (16.9%), fatigue (14.1%), leucopenia (9.9%) and transaminitis (8.4%). The combination of ICIs and anlotinib was safe and well tolerated, with no additional toxicity compared with monotherapy.

## Discussion

The synergistic effect of immunotherapy and antiangiogenic therapy for advanced NSCLC has been reported in many studies. In the studies of the IMPOWER150 and ORIENT31, the efficacy of ICIs + antiangiogenic drugs + chemotherapy and ICIs + chemotherapy was compared. Recently, several clinical studies have been underway to assess chemotherapy-free survival strategies, particularly as first- or second-line treatments. However, the later-line efficacy of ICIs plus antiangiogenic chemo-free therapy after EGFR-TKI progression was unknown. Therefore, we conducted a study of ICIs combined with anlotinib to explore the efficacy and safety in EGFR-TKI resistant population of this regimen.

Regarding whether this chemo-free antiangiogenic agent plus ICIs regimen could become the preferred later-line option for patients in whom EGFR-TKIs fail, our study provides important contributions to the evidence on the efficacy and safety of this combination regimen in previously treated patients with EGFR-mutant advanced NSCLC. We found patients with EGFR-TKI resistance had appreciable OS (mOS, 17.1 months) and the median PFS reached 5.8 months. EGFR-mutant patients with brain metastases achieved encouraging levels of tumor remission with this regimen (iORR: 38.5%, iDCR: 80.8%). The objective response rate (ORR: 19.7%) was not considerable, which may be related to the number of patients' previous treatment lines (median fourth-line), but the DCR could reach 77.5%. Compared with previous reports [22, 23], the incidence and severity of TRAEs were consistent and no new safety signs emerged with our ICIs plus anlotinib combination regimen. Grade 1 or 2 toxic effects were common, and only five patients discontinued treatment due to adverse events. This combination therapy also resulted in slightly increased incidences of asthenia, transaminitis and hypothyroidism, possibly attributable to the overlap of the profiles AEs induced by ICIs and anlotinib. Regarding hematological toxicity, grade 3 or higher hematological toxicity associated with our combination regimen was less than that associated with combined chemotherapy. Thus, ICIs combined with anlotinib showed long-lasting efficacy and good tolerance in previously treated EGFR-mutant NSCLC patients.

Patients who bear EGFR mutations have shown limited clinical benefit from immune checkpoint inhibitor monotherapy in the second-line or later setting [24, 25]. The PI3K-Akt, MAPK, and NF-kappa B signaling pathways and AP-1 are involved in the upregulation of PD-L1 induced by different EGFR-TKI resistance mechanisms that promote immune escape in lung cancer [26]. These findings indicated that EGFR-TKI resistant patients with increased tumor PD-L1 expression could exhibit improved responses to immunotherapy. ATLANTIC is a phase 2, open-label, single-arm trial that assessed the effect of durvalumab as a third-line or later treatment for advanced non-small cell lung cancer in patients with EGFR mutations or ALK rearrangements. Although EGFR/ALK patients with PD-L1 expression ≥ 25% had a relatively high response rate, the overall response rate was still lower than that in the wild-type population and could not translate to improved survival outcomes. The subgroup analysis of the ALTER0303 study demonstrated [19] that anlotinib is effective in both PFS and OS in EGFR-mutated patients with 5.6 mos of mPFS (95%CI 4.3–6.1) and 10.7 mos of mOS (95%CI 8.8–15.8). This favorable survival result provides evidence for the use of anlotinib as later-line therapy after EGFR-TKI resistance. Our combination therapy of anlotinib with ICIs further resulted in a mOS benefit of 6.4 months compared to the EGFR-mutated patients in the ALTER0303 trial who did not receive co-immunotherapy (mOS:17.1mos vs 10.7mos). In addition, a recent study [16] reported that anlotinib had a latent synergistic antitumor effect with ICIs by optimizing antitumor innate immunity. The modest but potentially promising benefit of adding anlotinib to immunotherapy is suggested for patients with a high unmet medical need.

The previous anlotinib combined ICIs therapy in EGFR-TKI resistant patients showed excellent survival with mPFS 4.3 mos and mOS 14.2mos [27]. The result of our survival is slightly higher than that study, which may be due to the relatively small number of patients were combined in later-line therapy (59.2% vs. 73.7%) and included patients (25.4%) in combined chemotherapy. However, due to the small number of samples included in previous study, it also affects the reliability of the results to a certain extent. Chen et al. prospectively explored the third- or further-line efficacy of EGFR ex20ins mutation, presenting a conspicuous 20mos of mOS [28]. The result of our rare mutation showed 12.6 months (95%CI 8.5–16.8), and the total EGFR population was 17.1 months (95% CI 12.0–22.3). However, this considerable OS survival result may be correlated with the further treatments after ICIs plus anlotinib. In our study, 81.7% of patients with ECOG PS0-1 was in better physical condition and received combined ICIs combined with chemotherapy plus antiangiogenic agents, or enrolled in clinical trials of new drugs or patients with oligometastatic further augmented radiotherapy.

Different EGFR mutations affect the immunogenicity of the TME and the response to immunotherapy [29]. In our study, we observed that the type of EGFR mutation may be associated with different survival outcomes. Although the difference was not statistically significant, our study suggested that, in contrast to patients with EGFR 19del subtype, patients harboring EGFR 21L858R mutation may benefit more from combined immunotherapy; moreover, Thr790Met-negative, but not Thr790Met-positive, patients showed survival benefits in terms of PFS and OS (PFS: *p=* 0.003, HR= 0.436, 95% CI 0.253–0.752; OS: *p* = 0.001, HR = 0.360, 95% CI  0.192–0.673). These findings suggest that we can further explore the roles of different components of EGFR tumor biology, such as EGFR mutation types, and that they may predict the response to ICI combined therapy. Furthermore, consistent with previous reports, patients who harbored the EGFR 21L858R mutation had a greater response rate than patients who harbored the EGFR 19del mutation. Mazieres J et al. showed that patients with the EGFR 21L858R mutation had a better prognosis than patients with the 19del mutation after immunotherapy. According to the IMMUNO-TARGET registry study, PFS significantly differed across EGFR molecular subgroups: 1.8 months for exon 19 and 2.5 months for exon 21 (*P* < 0.001) [30]. Mechanistically, patients with the 21L8585R mutation have a greater tumor mutation burden (TMB) than patients with the 19del mutation [31]. Moreover, Jin R et al. [9] reported that a greater proportion of patients with dual-positive hallmark (PD-L1 + /TIL +) disease were in the 21L858R-mutant group than in the exon 19del-mutant group (*P* < 0.05), suggesting that patients harboring the EGFR 21L858R mutation have an inflammatory phenotype with adaptive immune resistance and appear to benefit from anti-PD-1/PD-L1 immunotherapy. Additionally, a second interim analysis of ORIENT-31 [12] revealed that NSCLC patients with Thr790Met-negative or EGFR exon 21 Leu858Arg mutation were more likely to benefit from combined immunotherapy than those with a Thr790Met-positive or EGFR exon 19 deletion. Several preclinical studies have reported that PD-L1 expression in Thr790Met-negative individuals is higher than that in Thr790Met-positive individuals after EGFR-TKI treatment [32, 33]. Recently, several translational studies have suggested that resistance to tyrosine kinase inhibitor treatment might confer a favorable tumor immune microenvironment for subsequent immunotherapy owing to an increase in PD-L1 expression in tumor and immune cells as well as the density of tumor-infiltrating lymphocytes [12, 33, 34]. Although the survival benefit of T790M-positive patients was inferior to T790M-negative patients in subgroup analysis, T790M-positive patients also achieved favorable survival results as a later-line regimen with 4.6 months of mPFS and 11.3 months of mOS. A recent study showed that patients with acquired EGFR-TKI resistance could benefit from anlotinib through the inhibition of FGFR1 [35]. Considering the synergistic effect of immune and antiangiogenic drugs, regardless of which type of EGFR mutation is involved in resistance to EGFR-TKIs, this combination could be used as a candidate treatment.

During the disease process, up to 40% of patients with NSCLC develop brain metastases, and patients with EGFR mutations have a greater risk of brain metastases (OR = 3.83, 95% CI = 1.72–8.55; *p* = 0.001) [36, 37]. Moreover, the treatment of BMs with TKI resistance remains difficult, and there are limited associated efficacy data for this EGFR mutant population. Thus, we focused on analyzing the curative effect in the brain metastasis subgroup. There was no significant difference in the intracranial response to anlotinib plus ICIs between patients with BMs and patients without BMs (mPFS: 5.6 months vs. 6.0 months, *p* = 0.423; mOS: 14.4 months vs. 20.5 months, *p* = 0.107); our median PFS was consistent with that observed in the brain metastasis subgroup analysis of Wang et al. [17] (mPFS: 5.8 months). Remarkably, it is surprising that the ORR (38.5%) and DCR (80.8%) were considerable as a posterior-treatment regimen for brain metastases with EGFR mutations. A post hoc analysis of the phase 3 ALTER0303 study revealed that anlotinib could play a role in tumor control at the intracranial site. The intracranial ORR was 14.3%, and the DCR was 85.7%, with prolonged PFS (HR = 0.29; 95% CI 0.15–0.56) and OS (HR = 0.72; 95% CI 0.42–1.12) in the anlotinib group compared with the placebo group [38]. Additionally, a phase 1b trial evaluated the use of sintilimab and anlotinib in the frontline treatment of advanced NSCLC regardless of the PD-L1 expression level or TMB; this chemotherapy-free regimen exhibited encouraging efficacy. Notably, in that trial, all four patients harboring BMs achieved an intracranial CR, and three of them achieved an overall PR [14]. Taken together, these findings indicate that the synergistic effect of novel molecular targeted drugs that can penetrate the blood‒brain barrier (BBB) strongly combined with ICIs may improve the control of CNS metastases. The large-scale clinical trial and real-world data analysis of ICIs combined with anlotinib in EGFR-mutant patients with brain metastases are expected.

To our knowledge, the standard treatment after EGFR-TKI resistance is chemotherapy alone or in combination with antiangiogenic therapy, but after no more than 5 months at most, the patient will progress again. For patients who have not received chemotherapy before, this is not a small blow due to its efficacy and toxic effects. Considering that ORIENT-31 excluded patients who previously received systemic antitumor therapy other than EGFR-TKIs, our later-line chemotherapy-free regimen had an overall survival benefit that was not inferior to that of the quadruple regimen of ICIs + antiangiogenic drugs + chemotherapy. This approach provides a better choice for patients who were not well treated or refuse chemotherapy after previous TKI progression. Thus, this retrospective study that provides real-world evidence regarding the later-line efficacy of anlotinib combined with anti-PD-(L)1 in patients with EGFR mutations after TKI treatment failure could be regarded as meaningful. However, our study has several limitations. The relatively small sample size and single-center retrospective study are the major limitations. And selection bias may exist due to the lack of a control group. The expression of PD-L1 in a substantial proportion of patients is unknown due to incomplete medical records, which prevents accurate evaluation of the predictive value of PD-L1 in this combined regimen. Therefore, a prospective clinical trial or real-world multicenter study of ICIs and anlotinib is needed. Future clinical trials such as NCT04239443 that include NSCLC patients with EGFR-TKIs progression might yield further clinical evidence. Moreover, how to accurately identify the beneficiaries, the most appropriate mode and dose of immunotherapy plus antiangiogenic agents in treating advanced EGFR-mutated NSCLC patients represent issues that need to be further explored.

## Conclusions

In our retrospective study, we found that the later-line regimen of an anti-PD-1/PD-L1 antibody plus anlotinib yielded a promising survival outcome in NSCLC patients with EGFR mutations. We also observed that this combined regimen had good antitumor efficacy in patients with brain metastasis. Although our study provides evidence for the combination of ICIs and antiangiogenic agents in the EGFR-mutant population, determining a suitable dosage of antiangiogenic drugs and appropriate biomarkers for predicting survival are problems that must be explored before application in clinical practice.

### Supplementary Information

Below is the link to the electronic supplementary material.

Supplementary file1 (DOCX 20 kb)

## Data Availability

The datasets used and/or analyzed during the current study are available from the corresponding author upon reasonable request.

## Abbreviations

AE: Adverse event
BBB: Blood brain barrier
BM: Brain metastases
CI: Confidence interval
CNS: Central nervous system
CR: Complete response
CT: Computed tomography
CTCAE: Common terminology criteria for adverse events
DCR: Disease control rates
ECOG-PS: Eastern cooperative oncology group performance status
EGFR: Epidermal growth factor receptor
FGFR: Fibroblast growth factor receptor
HR: Hazard ratio
ICI: Immune checkpoint inhibitor
iCR: Intracranial complete response
iPD: Intracranial progressive disease
iPR: Intracranial partial response
irAE: Immune-related adverse event
iSD: Intracranial stable disease
LUAD: Lung adenocarcinoma
MRI: Magnetic resonance imaging
NSCLC: Non-small cell lung cancer
ORR: Overall response rate
OS: Overall survival
PD: Progressive disease
PD-1: Programmed death-1
PDGFR: Platelet-derived growth factor receptor
PD-L1: Programmed cell death-ligand 1
PFS: Progression-free survival
PR: Partial response
RECIST: Response evaluation criteria in solid tumors
SD: Stable disease
TKI: Tyrosine kinase inhibitor
TMB: Tumor mutation burden
TME: Tumor microenvironment
TRAE: Treatment-related adverse event
VEGFR: Vascular endothelial growth factor receptor
