# Supplementary material for: The later-line efficacy and safety of immune checkpoint inhibitors plus anlotinib in EGFR-mutant patients with EGFR-TKI-resistant NSCLC: a single-center retrospective study
Source: Cancer Immunol Immunother. 2024 May 17;73(7):134. doi: 10.1007/s00262-024-03712-7 (PMC11101402; doi:10.1007/s00262-024-03712-7)
Supplement: Supplementary file 1 — Supplementary file1 (DOCX 20 kb) [file 262_2024_3712_MOESM1_ESM.docx]

**Table S1 Multivariate Cox Regression Analysis of Factors Associated with PFS and OS**

| **Variables** | **PFS** | | **OS** | |
| --- | --- | --- | --- | --- |
|  | **HR (95% CI)** | **p value** | **HR (95% CI)** | **p value** |
| Gender  Male Vs Female |  |  |  |  |
| Smoking status  Never smoke Vs smoker |  |  |  |  |
| ECOG-PS  0-1 Vs 2 | 0.493(0.244-0.998) | **0.049** | 0.368(0.174-0.777) | **0.009** |
| Metastatic organs  <3 Vs ≥3 | 0.583(0.290-1.170) | 0.129 | 0.435(0.207-0.989) | **0.047** |
| T790M mutation  No Vs Yes | 0.623(0.337-1.152) | 0.131 | 0.580(0.274-1.228) | 0.155 |
| Type of mutation  Rare Vs 19Del  21L858R Vs 19Del |  |  | 0.948(0.397-2.262)  0.827(0.400-1.707) | 0.903  0.607 |
| Anlotinib dosage  12mg Vs 8mg  10mg Vs 8mg |  |  |  |  |
| Liver metastases  Absent Vs Present |  |  | 1.090(0.485-2.453) | 0.834 |
| Bone metastases  Absent Vs Present | 0.993(0.549-1.794) | 0.981 |  |  |
| Brain metastases  Absent Vs Present |  |  |  |  |
| Combined Chemotherapy  NO Vs Yes |  |  |  |  |

PFS, progression-free survival; OS, overall survival; ECOG-PS, Eastern Cooperative Oncology Group performance status: EGFR, epidermal growth factor receptor; T790M, Thr790Met; 21L858R, 21Leu858Arg; 19Del, Exon 19 deletion; HR. hazard Ratio; Cl, confidence interval; Bold values indicates significant results with p < 0.05.

**Table S2. Treatment-related adverse events with an incidence of at least 10% in study population**

|  | No. (%) of patients (n=71) | | |
| --- | --- | --- | --- |
|  | All grades | Grade 1-2 | Grade 3-4 |
| Fatigue | 43 (60.6) | 33 (46.5) | 10 (14.1) |
| Hypertension | 40 (56.3) | 28 (39.4) | 12 (16.9) |
| Transaminitis | 26 (36.6) | 20(28.2) | 6 (8.4) |
| Hypercholesterolemia | 27 (38.0) | 27 (38.0) | 0 |
| Nausea/vomiting | 23 (32.4) | 19 (26.8) | 4 (5.6) |
| Leucopenia | 22 (31.0) | 15 (21.1) | 7 (9.9) |
| Elevated blood thyrotropin | 21 (29.6) | 21 (29.6) | 0 |
| Hood-foot syndrome | 20 (28.2) | 17 (24.0) | 3 (4.2) |
| Hypertriglyceridemia | 19 (26.8) | 19 (26.8) | 0 |
| Decreased hemoglobin count | 18 (25.3) | 14 (19.7) | 4 (5.6) |
| Decreased appetite | 16 (22.5) | 13 (18.3) | 3 (4.2) |
| Diarrhea | 15 (21.1) | 12 (16.9) | 3 (4.2) |
| Rash | 14 (19.7) | 12 (16.9) | 2 (2.8) |
| Hypothyroidism | 11 (15.5) | 7 (9.9) | 4 (5.6) |
| Hemorrhage* | 10 (14.1) | 10 (14.1) | 0 |
| Thrombocytopenia | 10 (14.1) | 7 (9.9) | 3 (4.2) |
| Proteinuria | 9 (12.7) | 9 (12.7) | 0 |
| Headache/Dizziness | 7 (9.9) | 7 (9.9) | 0 |

*Adverse hemorrhagic events included hemoptysis, epistaxis, bronchial bleeding, gingival bleeding, gross hematuria, fecal occult blood and a case of brain metastatic tumor bleeding
